# Service Robots in Nursing Homes (SeRoNu): a holistic model of influencing factors

**DOI:** 10.1007/s11612-022-00639-4

**Published:** 2022-07-01

**Authors:** Lisa Obst, Franziska Bielefeldt, Rüdiger von der Weth, Michael Dick

**Affiliations:** 1 Hochschule für Technik und Wirtschaft Dresden, Dresden, Germany; 2 Otto-von-Guericke-Universität Magdeburg, Magdeburg, Germany

**Keywords:** Care crisis, Robot, Nursing Home, Pflegenotstand, Roboter, Stationäre Altenpflege

## Abstract

This article in the journal *Gruppe. Interaktion. Organisation. *introduces a model that provides an overview and orientation for science and practice regarding robots in elderly care. Aging societies and the lack of professionals working in elderly care put strain on the care sector in many countries worldwide. Robots can be a possible support for caregivers and assistance for people in need of care. However, their (future) usage comes along with various challenges and currently there are only few examples of use in practice. The data of the developed holistic triple-layered shell model *SeRoNu* (***Se***rvice ***Ro***bots in ***Nu***rsing Homes) is based on three conducted studies: (I) A *work analysis* (HTO-Approach; Strohm and Ulich 1997), (II) *future workshops* (Jungk and Müllert 1989) and (III) *expert interviews*. Social robot *Pepper* is used as an example of application, as the model offers a framework for different service robots. The article illustrates the influencing factors and the diversity of robotic solutions to the care crisis. As a result, a multi-professional approach is required as the different aspects need to be considered individually.

## Abstract (de)

Dieser Artikel in der Zeitschrift *Gruppe. Interaktion. Organisation. s*tellt ein Modell vor, welches einen Überblick sowie eine Orientierung für Wissenschaft und Praxis in Bezug auf einen Robotereinsatz in der Altenpflege geben soll. Alternde Gesellschaften und der Fachkräftemangel im Altenpflegesektor belasten viele Länder weltweit. Roboter können eine mögliche Lösung zur Unterstützung der Pflegekräfte und Assistenz für Pflegebedürftige darstellen, wobei deren Einsatz mit zahlreichen Herausforderungen und wenigen Vorbildern aus der Praxis einhergeht. Die Daten des ganzheitlichen dreischichtigen *SerosA-Modells* (***Se***rvice***ro***boter in der ***s***tationären ***A***ltenpflege) basieren auf drei durchgeführten Studien: (I) Eine *Arbeitsanalyse* (MTO-Analyse; Strohm und Ulich 1997), (II) *Zukunftswerkstätten* (Jungk und Müllert 1989) und (III)* Experteninterviews. Pepper* als ein sozialer Roboter dient hierbei als Anwendungsbeispiel des Modells, welches für verschiedene Typen von Servicerobotern gilt. Der Beitrag veranschaulicht die Einflussfaktoren und die Vielfalt robotischer Lösungen im Pflegenotstand. Ein multiprofessioneller Ansatz ist erforderlich, um die zahlreichen Aspekte individuell zu betrachten.

## Introduction

Currently,* care crisis* is a well-known buzzword worldwide. Due to demographic change that has caused an ageing population, as well as fewer employable people, societies face a huge shortage of professionals—especially in the care sector. A prognosis for Germany estimates that there will be a lack of up to 307,000 caregivers in 2035 (Flake et al. 2018).

To counteract the shortage of labour force and to ensure a high quality of care, different solution approaches exist—such as the recruitment of skilled workers from abroad or general improvements in working conditions (Theobald and Leidig 2018). A future solution to support care workers could also be the usage of *service robots* which “perform […] useful tasks for humans or equipment excluding industrial automation applications” (International Organization for Standardization 2021, para. 2.9) and therefore can fulfil various tasks in the service industry. As a consequence, several different (sub-)types and classifications of service robots exist (e.g. Čaić et al. 2018) as well as reviews about their application in elderly care (e.g. Abdi et al. 2018; Chen et al. 2018; Shishehgar et al. 2018). In view of this issue focusing on *social robots*, the application example of the later depicted model is limited to this sub-form which can be defined as follows:

[They] are designed to interact with people in human-centric terms and to operate in human environments alongside people. Many social robots are humanoid or animal-like in form […]. A unifying characteristic is that social robots engage people in an interpersonal manner, by communicating and coordinating their behaviour with humans through verbal, nonverbal, or affective modalities. (Breazeal et al. 2016, p. 1936)

A well-known example is child-like robot *Pepper *(SoftBank Robotics 2022), which will be used as an application example in 3.2. It is used in the elderly care context as well: “Its main role is to engage people […]: interactively provide information on a company’s offer, to greet and amuse customers, or to influence the prominence of a company” (Gardecki and Podpora 2017, p. 1). In general, *Pepper* is meant to increase quality of life of the elderly and relieve personnel in nursing homes (Takanokura et al. 2021).

Although the use of robots sounds promising in theory, it is complex to develop, finance, implement and successfully use them. As a result, the usage of robots has various consequences (e.g. work design, qualification) and issues (e.g. technology acceptance, ethics) as described by Friesacher (2010) and Zöllick et al. (2020). Therefore, in this article a model is introduced that aims to give an overview and orientation for science and practice regarding robots in elderly care. It is a framework derived from different explorative studies described in the following—a related explorative work with regard to robots in the care sector was published by Pijetlovic (2020).

The leading research question is: *Which aspects concerning the implementation and the usage of service robots in an inpatient elderly care facility are considered relevant by stakeholders and can be identified after an integrative data analysis?*

## Methodology

As the developed model is based on data of three explorative mixed-methods studies, they will be briefly described in 2.1. After giving an overview about the data base, the procedure of model conception is particularized in 2.2 to exemplify how the various factors and layers have been derived.

### Data base

**Fig. 1:**
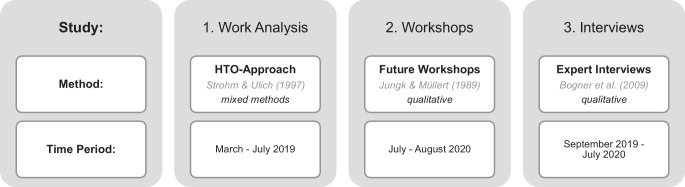
Studies and Survey Procedure

The three main studies, which are summarized in Fig. 1, have been conducted to gain data from a nursing home and various stakeholders in order to portray different perspectives on the (potential) usage of service robots in nursing homes. They are briefly described in the following paragraph and were partly described in previous publications (e.g. Bielefeldt 2020; Bielefeldt et al. 2020; Obst et al. 2020).^1^

In the first study, a holistic psychological work analysis within a nursing home has been conducted to fully understand the care facility and the cooperating residential unit (e.g. culture, history, processes, tasks, attitudes). The *HTO-Approach* by Strohm and Ulich (1997) is one possible method of (psychological) work analysis that considers the relevant components *organization, humans* and *technology *and is originally characterized by seven levels of research. All of them have been realised in an adapted mixed-methods design with a different methodology on each level (e.g. expert interviews with executives, surveys with employees, document analysis) so that the sample size varies (*n* = 2–19).

In the second study, the qualitative method *future workshops* was conducted within the same nursing home. In general, *future workshops* are described as a social problem-solving procedure and allow their attendees to participate regardless of their personal or social backgrounds (Müllert 2009). In this study, the residential units’ staff (*n* = 8) discussed robot usage in nursing homes. Jungk and Müllert (1989) defined three phases the participants are accompanied through: (I) *Critique*, (II) *Fantasy* and (III) *Implementation Phase*.

In the third study, fifteen *expert interviews *(e.g. Bogner et al. 2009; *n* = 18) with various stakeholders (e.g. nursing home management, works council, health and nursing care insurance, robot manufacture*r*, nursing scientist, ethicist) were conducted. All stakeholders were interviewed as representatives of their institutions and had different prior knowledge of both research objects, *elder care *and *robots in elder care*. The applied interview guideline contained a variety of questions about attitudes towards service robots in nursing homes (e.g. potentials and risks of their usage, desired and rejected robot functions).

### Model conception

**Fig. 2:**
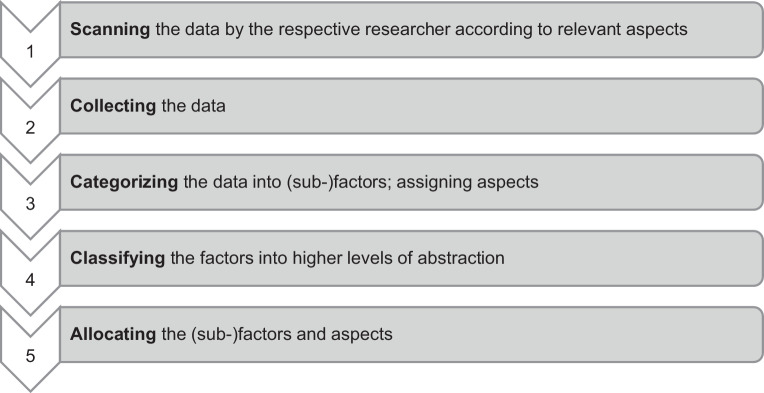
Procedure of Model Conception

The factors underlying the model were derived *inductively* from the presented three studies in 2.1. Due to the mixed-methods approach, both qualitative and quantitative data are incorporated in the model. The procedure for the development of the model is shown in Fig. 2.

First, the existing data from the three studies was *scanned* by the two researchers and searched for relevant aspects according to the usage of robots in nursing homes (1). Then the statements and information that were considered relevant according to the implementation and usage of robots in elder care were *collected* in a table (2). A mind map was then created from this collection of data, which was used as a structuring aid—data with similar content could be *categorized* in this particular way. In addition, the mind map summarised aspects with similar content and formulated initial factors (3). After the data had been collected, structured and categorized into (sub-)factors, a *higher level of abstraction* was necessary for classifying these. The model by Mütze-Niewöhner and Nitsch (2020) was chosen as a suitable reference frame and its structure was adapted to suitable layers for the model which assembled the given multitude of (sub-)factors. The structure consisting of the inter-company, company level and the core work tasks was adopted (4). Ultimately, the (sub-)factors and underlying aspects which were derived from the data were *allocated* to the fitting level. Some factors were classified across various levels, as they were considered particularly relevant throughout the shells of the model. A table that was used in the development of the model was also structured per the model levels. It included all factors/sub-factors, the description of the aspects and the respective information on the data source (5).

## Results

**Fig. 3:**
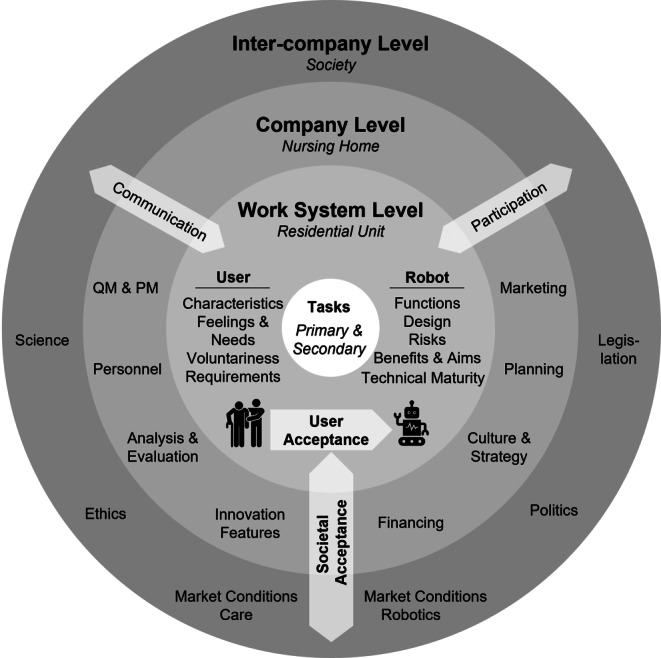
SeRoNu-Model (*Se*rvice *Ro*bots in *Nu*rsing Homes; user icon: Flaticon.com)

A shell model called *SeRoNu* (***Se***rvice ***Ro***bots in ***Nu***rsing Homes; Fig. 3) was derived. It consists of three layers: (I) *Inter-company level*, (II) *Company level* and (III) *Work system level*. Tasks for elderly care make up the core of the model. Each level contains (sub-)factors, which can affect and can be affected by the use of service robots in nursing homes. In the following section, *SeRoNu* will be described (3.1) and applicated (3.2).

### Model description

The holistic model aims to provide a comprehensive representation and an overview of factors, that might be relevant and are worthy of consideration for different stakeholders, such as nursing home staff or strategic decisionmakers in care. Thus, the model addresses researchers and practitioners. For example, its intent is to provide an overview of relevant aspects for nursing home managers, who are considering purchasing a robot. Furthermore, it is the first model that sums up relevant constructs and aspects that have been investigated separately for the specific type of *service robots* in the specific context of *stationary elderly care*. Each level of *SeRoNu*s’ composing factors (and subfactors) will be explained briefly.

#### Inter-company level

The inter-company level contains factors on a societal level, which goes beyond the influence of a company. However, it can exert influence on the implementation of robots in nursing homes:

*Science.* Research on robots comes with challenges, e.g. there is competition for research funds and often developments do not make it into practice. There is also a desire for interdisciplinary research that also involves users.

*Market Conditions: Robotics.* For manufacturers, the robot’s development for inpatient elderly care is very complex and is cost- and time-intensive. Multifunctional robots that can be used in different areas/sectors are found more attractive from the manufacturer’s point of view.

*Market Conditions: Care.* The market environment for care is characterised by competition for skilled workers. Robots could influence competition between facilities through effects on the quality of care and/or the price.

*Legislation (Data protection, Liability).* Data protection is a topic that is considered particularly relevant. It covers various aspects—the collection, storage, access, use and general security of data. Liability issues are relevant for stakeholders and have not yet been legally clarified from their perspective. It is unclear who is accountable for any damage that could be caused by the robot, because robots themselves cannot be held liable. There may also be damages to the robot that someone will be required to pay for. In addition, there are various laws and regulations that may be applicable regarding the use of robots.

*Politics (Political Guidelines, Financial Support).* In recent years, various laws have been created that have brought changes in inpatient elderly care in Germany. Politics could also influence the development of robots in care, in particular by providing research funds and financial support. However, funding opportunities are limited. In the long term, robots would have to be included in SGB XI (regulations of the social long-term care insurance) in order to be financed.

*Ethics. *Another relevant topic is the question of ethical compatibility of various applications of robots with respect to users (residents and employees). In principle, the use of robots in secondary activities (e.g. documentation) is often considered less objectionable.

To summarize, the range of factors on the inter-company level emphasize the diverse scope for development and use of robots in care lying in society. Robots can only be used successfully in nursing homes if the appropriate framework conditions are in place.

#### Company level

The middle level of the model is the company level, which concerns the management of a nursing home:

*Innovation features. *An innovation, such as the use of a robot in an inpatient care facility, is characterised by several features. One is the underlying trigger and motive of the innovation. Another is the extent of the innovation and the actual novelty of the robot. Furthermore, the temporal scope of the process characterises the innovation.

*Planning (Implementation Planning, Technical and Spatial Requirements). *When planning a robot deployment, there are various aspects that should be considered: Objectives & benefits of the robot, responsibilities, pilot run possibilities, project planning, legal certainty, existing expertise, information & decision paths, and promoters & multipliers. In addition to the basic technical requirements, structural conditions (e.g. sufficient space in corridors and residents’ rooms) should be suitable for the use of robots.

*Personnel (Qualification, HR Management).* Depending on the type of robot, there are different qualification requirements, that range from short instructions for individual employees to extensive training of all employees. There is a wide variety of effects on human resource management (e.g. changes in gender ratio, fluctuation, working time models) that could be expected as well.

*Analysis & Evaluation. *Various analyses should capture the situation before the robot is used. For example, a team, technique profile or a load and stress profile can be created. It is recommended to repeat the analyses after the robot deployment, in order to evaluate the success of the project.

*Quality & Process Management. *In quality management, adjustments may be necessary in standards, documents or the organisation chart. Effects on quality improvement, certifications and possibly on audit regulations should be assessed. The robot also imposes demands on process management. Processes need to be recorded before the robot is used and re-planned for its introduction. After the introduction of the robot, processes should be further reviewed and must be kept in view.

*Financing. *Federal funding opportunities and funding opportunities including health and long-term care insurance funds should be examined by the facility. If these are not available, the robot must be financed by the facility’s own financial resources. The expected usage time, utilisation and amortisation time should be considered, as well.

*Marketing. *The robot can be a unique selling point of an institution and therefore could be used for marketing purposes.

*Corporate Culture & Strategy. *A comparison of the corporate values and the corporate philosophy with the use of robots should be carried out. The robot should also be included in strategic considerations.

In conclusion, the multitude of factors on the company level indicate, that the use of robots in nursing homes requires extensive preparation in various areas within the nursing home as a company.

#### Work system level

The innermost level of the shell model is the work system level. It represents a residential unit of a nursing home in which *users* (staff & residents) would interact with a *robot*:

#### *User*

*Characteristics. *Each resident’s personal needs regarding their disease pattern, abilities, and previous experiences with technology should be considered. Employees should also be individually considered based on their previous experience and attitudes towards robots.

*Feelings & Needs. *Various feelings can be triggered by the use of the robot, including fear. The possible influence that the robot may have on the user’s individual needs must also be examined.

*Voluntariness. *Self-determination of employees and residents regarding the use of robots is desirable but can only be implemented to a limited extent depending on the type of robot.

*Requirements. *Depending on the type of robot, different work demands may be placed on employees. Control and supervision of the technology use by employees will be necessary to varying degrees. Technological failures should be dealt with as well as additional tasks instead of ones that are conducted by the robot.

#### *Robot*

*Functions. *Different functions can be performed by different types of robots. In addition to specific main functions, robots may have speech recognition and possibly emotion recognition as well as sensors.

*Design. *Relating to the design of robots, there are many possibilities. The appearance can be designed to resemble a human or an animal, for example. There are also different design options in terms of size, language and voice of the robot.

*Benefits & Aims. *Depending on the type of robot and its functions, robots can provide different benefits. Often, the main goal is to support and relieve employees. Mainly, physically and mentally demanding work should be taken over. Robots also provide benefits with the removal of routes at work and documentation. There are also benefits for the residents in therapy and care services, or increased safety.

*Risks. *Risks of using robots can consist of various safety issues. A risk of fragmentation and clustering of care processes is possible. Another concern is that this could lead to additional workload for employees, job loss, or that robots will ultimately take centre stage at the nursing home instead of human care.

*Technical Maturity. *At the moment, there are still few technically mature care robots in the market. Quality features could be: practicability, durability, sensitivity, multifunctionality, compatibility or reliability.

Consequently, the factors on the work system level indicate how robots should be created and behave in order to be accepted by the users in accordance with their individual characteristics, experiences, feelings and needs.

#### Core-tasks

Tasks related to care are the core of the model. They can be divided into *primary tasks*: nursing (e.g. personal hygiene), care (e.g. companionship), supply (e.g. food) and therapy. *Secondary tasks *(e.g. documentation) do not take place in direct contact with the residents.

#### Additional factors

Besides the model factors that were previously mentioned, there are three factors which overlap different levels of *SeRoNu*. They are relevant and especially important on all levels when taking into consideration the usage of robots in nursing homes.

*Communication*. Across all levels, communication is important—from the communication of research results to the public, to the communication with employees, residents and their relatives in a facility.

*Participation*. On one hand, participation should be possible for users in the development of the robots. Care representatives should also be able to participate in policy and committee decisions regarding robotics. In the institution itself, participation should be enabled in the decision to deploy a robot, while planning the design of its use, and in its evaluation.

*User & Societal Acceptance*. The acceptance of robots by users and society is an important aspect. Users’ feelings can vary between enthusiasm for technology and fear/resistance. In addition, previous experience with technology and personal preferences can have an influence on user acceptance. Depending on the type and design of the use of a robot, there are also different degrees of acceptance. In society, there often is a lack of information on how robots can be used in care, which initially leads to scepticism. However, robots are already used as a matter of course in industry. Ultimately, societal acceptance of robots is likely to increase when successful use of robots in care is observed. Simultaneously, user acceptance could be influenced by attitudes within society, especially by those of their relatives. Furthermore, acceptance can be viewed both as a prerequisite for and result of successful robot use.

### Social robot pepper in nursing homes: application example of the seRoNu-model

Subsequently, practical implications of selected factors of the model shall be illustrated with an application example. For this purpose, *Pepper *(introduced in 1.) will be used to exemplify three (sub-)factors. *Pepper *is a child-like robot used mainly for social interaction and entertainment.

From an *ethical* perspective, an assessment of *Pepper* can be seen critically. It is used for communication, entertainment or even in therapy in close contact to its users. Furthermore, the users are mainly the elderly who are more vulnerable due to physical or cognitive restrictions. Therefore, the amount of interaction should be considered when deciding whether the use of *Pepper* reduces the duration of communication with humans (e.g. staff) or increases it (e.g. video calls with relatives). Questions of transferring responsibility in dangerous situations or even possible terminal care by this robot are less urgent, since it does not take on any fully autonomous or life-critical tasks at the moment.

At the *company level, *there are *planning* requirements for the use of *Pepper* because the robot needs a barrier-free environment, WIFI-access and power supply/battery load for instance. Due to its child-like size the robot does not require a lot of space within the nursing home. Regarding *Personnel* requirements, extensive training might be necessary to operate *Pepper*. Basic knowledge about the robot’s functionality should be taught (e.g. about hardware and software components, data protection). *Pepper* is not capable of replacing care personnel with its given functions. Nor can it create other more drastic changes, like in rosters or working time models.

Both the *robot* and the *users* are part of the *work system level*. Pepper could fulfil different needs for both user groups (care personnel and elderly people): e.g. harmony, security, play and support. However, dignity, respect and humanity might be threatened by using *Pepper *in nursing homes. In addition, positive feelings such as excitement, balance, reassurance, fulfilment, joy or relief could occur. On the other hand, negative feelings such as suspicion, fear, inhibition or insecurity could also arise. Concerning *benefits & aims* of the usage of *Pepper* within nursing homes, it is mainly aimed to the robot entertaining and activating the residents. If people in need of care are experiencing positive effects, then employees could indirectly benefit from the robot as well (Takanokura et al. 2021).

## Discussion

Social robots such as *Pepper* can offer various advantages in nursing homes, but their use also comes with challenges. The *SeRoNu-Model* shows that a variety of factors are relevant for the use of robots in elderly care facilities. When looking at the implementation factors and aspects that are considered worthy of attention, it becomes apparent that they’re not fundamentally different from the introduction of other technical innovations in companies such as software (e.g. requirements on participation and qualification) (Zerth et al. 2021). However, depending on the specific model of the robot, the requirements or changes may be more profound than for other technical innovations found in nursing homes. The robot might have a bigger impact on aspects such as work organization than the implementation of an electronic nursing documentation or lifting aids. Robot’s special features are their ability to work autonomously and the areas in which they could potentially intervene. This may lead to fewer predictable actions (e.g. for residents) as is the case with manually operated aids. Furthermore, the autonomy allows the robot to fulfil more complex tasks (e.g. therapy components) which can cause a change in the personnels’ work routine.

Stakeholders that participated in the three studies didn’t show a general rejection of robots in care. In fact, they were rather open and mostly prevailed justified scepticism about certain aspects. They attributed great relevance to financing, since funding of robots still must be provided by the facilities and is not yet covered by nursing or health insurance in Germany. Data protection is also considered particularly critical by stakeholders with robot use (Radic and Vosen 2020). There also is an ethical debate about the topic, as shown by the extensive publication of the German Ethics Council (Deutscher Ethikrat 2020). Ethical concerns with the use of robots in elderly care could be the possibility for deception and infantilizing of elderly people or a reduction of human contact (Sharkey & Sharkey 2012).

There should always be a possibility to openly discuss whether there are alternatives for the use of robots in elderly inpatient care because robots are not regarded as *the* only solution to problems in nursing care. Human personnel are certainly preferable to robots, especially within interaction work, which forms the core of nursing care (e.g. Böhle 2011). If sufficient personnel would be available again, a trade-off between robots and human caregivers could take place under different preconditions.

However, the care sector is an industry whose framework conditions are constantly changing. Before the COVID-19 pandemic, the nursing situation had already become a greater focus of interest in society. Politically this has also been taken into effect in Germany, by recently introducing various legislative changes (e.g. Pflegepersonalstärkungsgesetz, Pflegeberufereformgesetz), that have affected the organization of nursing care facilities while trying to counteract the care crisis.

## Limitations and implications for further research

Since the data of the model was collected in an exploratory case study and mostly qualitative surveys, it cannot be considered representative for other use cases of service robots in nursing homes. Thus, further validation studies are necessary. Future research should aim to replicate and validate results and factors of the model, as well as to transfer the model into further contexts such as home care, rehabilitation, or hospitals. Robots in logistics and cleaning are already being used frequently in hospitals and various robots can be used in rehabilitation (Dahl and Boulos 2014).

A holistic, exploratory approach was taken, which did not limit the study to a specific robot type such as *Pepper *or the prediction of specific outcomes of a construct such as user *acceptance*. It is important to note the diversity and variety of data collected, that feeds into the integrative model. The model includes explicit knowledge and aspects considered relevant by interviewees, implicit data (derived from general aspects of technology introduction), as well as opinions, thoughts, and feelings. Thus, the model contains factors influencing the use of robots as well as design dimensions. Moreover, residents of a care facility have not been interviewed directly, the studies put their emphasis on the employees—a review has shown that they are not deeply involved often (Haubold et al. 2020). Finally, future research needs to evaluate the coding scheme to ensure reliability and validity.

In general, there are only a few tools such as questionnaires or workshops that are specifically geared towards robots in care. Scientifically proven models such as the Technology Acceptance Model (TAM) and its further developments (e.g. Davis 1989; Venkatesh and Davis 2000; Venkatesh and Bala 2008) examine one specific construct and do not provide a widespread view on this complex topic. Nevertheless, researchers such as Bröhl et al. (2019) have been building on the validated models (TAM 1–3) and adapted them to human-robot collaboration. However, their model focusses on industrial robots and therefore does not take into account the specific requirements that prevail in the context of nursing care.

## Conclusion and transfer

The *SeRoNu*-Model can be used to deliver an overview of the issues that should be taken into consideration when looking at the use of robots in nursing care. Nursing management might be able to focus on relevant factors such as data protection/data regulations, financing opportunities and the search for information on these topics. Furthermore, core tasks should be carefully examined in order to determine which tasks could be taken over by a robot and which of them should remain in human responsibility. The concept of individuality should also be emphasized. The individuality of residents and employees needs to be considered as well as the fact that each facility is different in terms of goals, capital resources, and technical equipment. Residents and employees are distinct in terms of their characteristics and preferences, but also their abilities.

In summary, the presented model emphasises the importance and diversity of possible robotic solutions against the care crisis and gives initial concrete recommendations for action. As a result, a multi-professional approach involving various stakeholders is required in order to develop technically mature robots for this sensitive application context.

## Footnotes

1 For more information see the dissertation of Franziska Bielefeldt and Lisa Obst (Title: Servicerobotereinsatz in der stationären Altenpflege: Explorative Analyse relevanter Faktoren unter Berücksichtigung diverser Stakeholderperspektiven sowie arbeitswissenschaftlicher Analysen eines Unternehmens dieser Dienstleistungsbranche) which was submitted in Dezember 2021 and will presumably be published in summer 2022.
